# Erbium emission in Er:Y_2_O_3_ decorated fractal arrays of silicon nanowires

**DOI:** 10.1038/s41598-020-69864-5

**Published:** 2020-07-30

**Authors:** Maria Josè Lo Faro, Antonio Alessio Leonardi, Francesco Priolo, Barbara Fazio, Maria Miritello, Alessia Irrera

**Affiliations:** 1 Dipartimento di Fisica e Astronomia “Ettore Majorana”, Università Di Catania, Via Santa Sofia 64, 95123 Catania, Italy; 2 CNR-IMM, Istituto per la Microelettronica e Microsistemi, Via Santa Sofia 64, 95123 Catania, Italy; 3 CNR-IPCF, Istituto per i Processi Chimico-Fisici, V.le F. Stagno D’Alcontres 37, 98158 Messina, Italy

**Keywords:** Optical physics, Techniques and instrumentation

## Abstract

Disordered materials with new optical properties are capturing the interest of the scientific community due to the observation of innovative phenomena. We present the realization of novel optical materials obtained by fractal arrays of silicon nanowires (NWs) synthesized at low cost, without mask or lithography processes and decorated with Er:Y_2_O_3_, one of the most promising material for the integration of erbium in photonics. The investigated structural properties of the fractal Er:Y_2_O_3_/NWs demonstrate that the fractal morphology can be tuned as a function of the sputtering deposition angle (from 5° to 15°) of the Er:Y_2_O_3_ layer. We demonstrate that by this novel approach, it is possible to simply change the Er emission intensity by controlling the fractal morphology. Indeed, we achieved the increment of Er emission at 560 nm, opening new perspectives on the control and enhancement of the optical response of novel disordered materials.

## Introduction

Silicon photonics is one of the most interesting topic in the field of integrated optics^1^. The push in this sector is closely linked to the compatibility with the microelectronics technology and the consequent realization of low cost circuits capable of implementing a great variety of functions in the same chip. The development of the microelectronics manufacture has made possible the production of microprocessors consisting of hundreds of millions of components packaged in dimensions of the order of a few cm^2^. The length of the metallic interconnections is now of the order of 10 km in a micrometric area and therefore many levels of metal are required. This dramatic increment of the total length of the metallic circuitry leads to a consequent increase of signal delays, signal cross-talks, and power dissipation, which are crucial limitations for further developments. The entire scientific community defines this problem as an electronic bottleneck, making the further reduction of the minimal feature size a very hard task^2^. The substitution of electrical signals with optical ones for information transmission and processing can definitely solve the signal delay time and power dispersion problems^3,4^.

The use of silicon-based optoelectronics could possibly solve many of these difficulties. However, bulk silicon has an indirect energy band gap and it is therefore highly inefficient as a light source. Many efforts have been focused towards the research of different strategies to solve the physical inability of Si to emit light at room temperature. The quantum confinement is the more promising approach to obtain light emission from silicon at room temperature. The most encouraging silicon confined systems are Si nanoclusters^5,6^, porous Si^7,8^ and in recent years, Si nanowires (NWs) have attracted increasing interest for photonics applications^9–11^. We already demonstrated the realization of Si NWs with an extremely small diameter, compatible with the observation of quantum confinement effects leading to the observation of light emission at room temperature. These Si NWs are obtained by using a modified metal-assisted wet etching process^10^, a preparation methodology that is at low cost, fast and compatible with silicon technology. Moreover, the study and optimization of their preparation allowed to realize arrays of Si nanowires with fractal geometry^12^, showing very interesting optical properties.

Fractals are spontaneously formed in nature in order to minimize the surface energy of the system, maximizing its entropy at the same time. Fractal structures are of great interest for the scientific community due to their extraordinary property of scale invariance able to promote light localization^13,14^ and non-linear optics effects^15,16^, combined to a strong electrical conductance^17,18^ and super-diffusion^19^. Due to the remarkable fluctuation of the refractive index across its morphology, fractal NWs are particularly suitable and promising for strong scattering and light localization effects. In particular, these fractal NWs show an efficient light trapping due to the in-plane multiple scattering^12^, which allows an enhancement of the system optical response as a function of the fractal parameters with potential for both photovoltaics and photonics applications. Among the efforts of the scientific community to efficiently produce photons from silicon, a very studied approach is the introduction of light-emitting impurities, such as rare earth ions. Among the rare earths, erbium is one of the most studied and interesting systems with the relevant advantage that standard silicon technology can be used to introduce erbium as a dopant in Si compatible material^20,21^. However, due to its low solubility and low excitation cross section, various approaches have been used to enhance the optical performance of Er^3+^-doped SiO_2_^22^. Some of these methods include the use of Er^3+^-sensitizers, such as silicon nanoclusters^23,24^ having an excitation cross section four orders of magnitude higher than that of erbium in silica. Moreover, Er can be also efficiently excited by non-resonant transfer. A crucial point that limits the erbium performances is the maximum concentration of Er^3+^ that can be inserted without the occurrence of clustering phenomena and non-radiative ion-ion interactions. The critical doping concentration depends on the matrix. Er tends to segregate and precipitate determining the erbium clusters formation that are optically inactive. The ion-ion interactions present another limit related to the high Er concentrations, which determine a decrease of the PL emission owing to the occurrence of these non-radiative phenomena^25,26^. The scientific community has focused lots of effort on the realization of Si compatible materials that allow the incorporation of high concentrations of optically active erbium ions, such as Er silicates^26–28^, or mixed compounds^25,29,30^. Among them, Y_2_O_3_ host has been emerged as an excellent compromise between the maximization of erbium concentration and the limitation of deleterious ion-ion interactions^31,32^. Indeed, since the crystalline structure of Y_2_O_3_ is identical to the one of Er_2_O_3_, the erbium ions can be introduced in the lattice of Y_2_O_3_ in Y substitutional positions, up to concentrations of 10^22^ at/cm^3^ without clustering^33,34^. Furthermore, the Y_2_O_3_ film is compatible with the silicon platform and can be synthesized with different industrial techniques, such as sputtering. We have created a new platform by coupling two materials with enormous potentialities in the photonics field with a low cost approach compatible with silicon technology. In fact, in this paper we decorated fractal arrays of Si NWs with Er-doped Y_2_O_3_ by an oblique sputtering process, tuning the fractal morphology according to the deposition angle and thus leading to an increment of the erbium emission.

## Results and discussion

### Decoration of silicon NWs with Er-doped Y_2_O_3_

Two-dimensional random fractal arrays of Si nanowires were prepared by metal assisted chemical etching of n-type Si (111) substrate (P-doped, ρ 1–5 Ω∙cm) by using 2 nm of Au discontinuous layer^35^. We decorated fractal arrays of vertically aligned Si NWs of about 2.5 µm length with Er-doped Y_2_O_3_ deposited by oblique angle magnetron sputtering. By this approach, we demonstrated that it is possible to obtain decorated fractal systems whose morphology can be varied as a function of the deposition angle.

**Figure 1:**
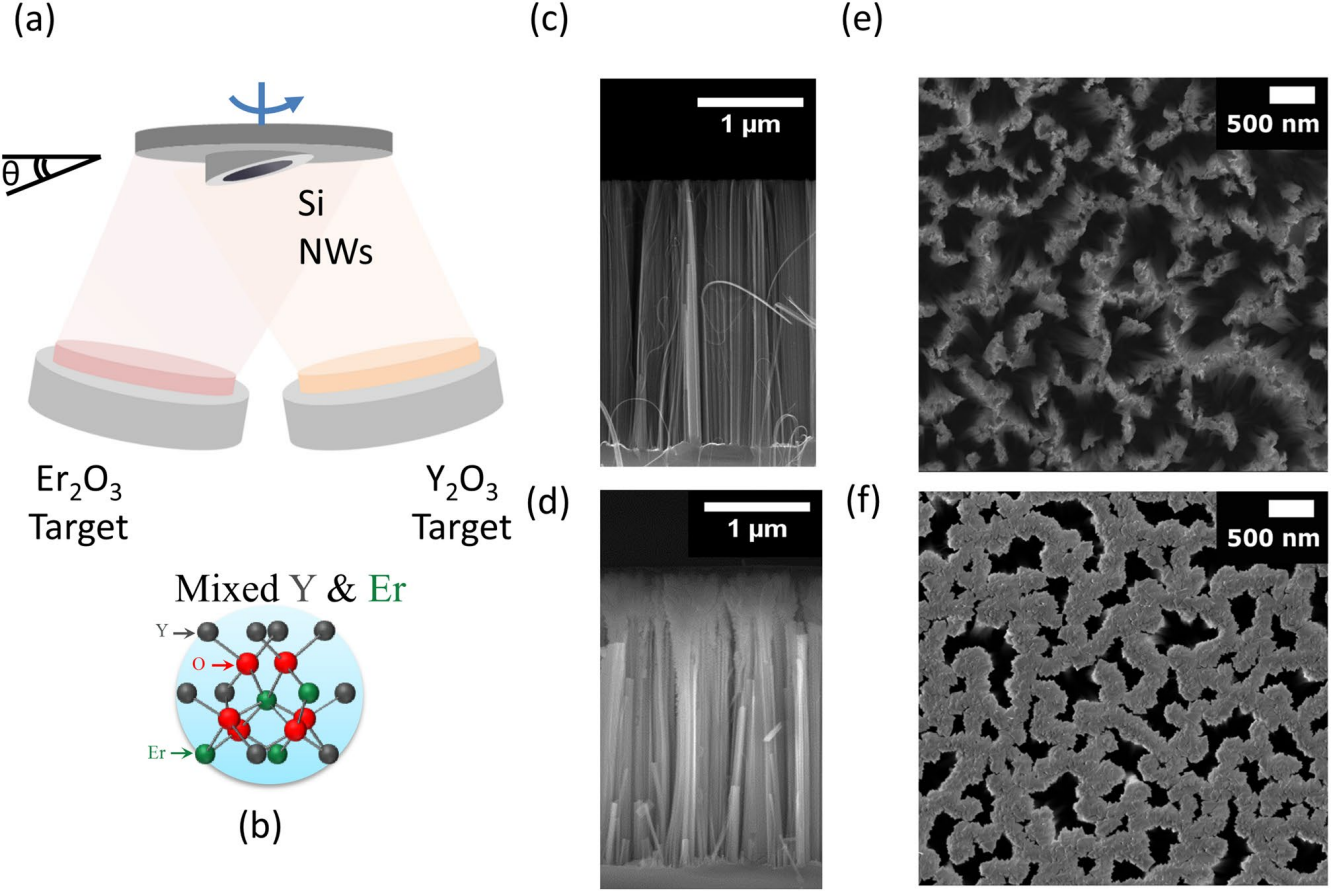
(**a**) Schematic of the oblique angle sputtering setup used for the decoration of Si nanowires arrays and a representative scheme (**b**) of the Er atoms occupying substitutional positions into the Y_2_O_3_ matrix. SEM microscopy of Si NWs arrays before and after decoration with Er:Y_2_O_3_ at an angle of 15° acquired in cross section (**c, d**) and in plan view (**e, f**), respectively.

A schematic illustration of the Er-doped Y_2_O_3_ decoration process of silicon NWs by oblique angle magnetron sputtering is depicted in Fig. 1a.

As-prepared NWs together with Si bulk reference substrates were allocated onto sample holders, each one tilted at a fixed angle θ with respect to the standard position parallel to the targets (θ = 0°). The Y_2_O_3_and Er_2_O_3_ targets were simultaneously co-sputtered in ultra-high vacuum magnetron sputtering (see “Material and methods” for details) on the rotating substrate holder heated at 300 °C. The deposition conditions have been optimized in order to synthesize a good quality Er-rich material, where all Er^3+^ ions are optically active into the yttrium oxide host, chosen also due to its Si-compatibility. Remarkably, Y_2_O_3_ and Er_2_O_3_ have the same crystalline cubic structure with similar lattice parameters, allowing the Er^3+^ to replace Y^3+^ ions in substitutional sites (as shown in the scheme in Fig. 1b). In this work, all the deposition parameters (with the exception of the tilt angle) were kept constant, thus guarantying the same stoichiometry and Er concentration for all the investigated samples. The scanning electron microscopy (SEM) cross section in Fig. 1c, d show the high density fractal array of vertically aligned Si NWs with length of 2.5 µm before and after the decoration at the angle of 15°, whose synthesis is described in the Methods section. The control of the oblique deposition angle onto vertical arrays of Si NWs permit to vary their morphology taking advantage of their self-shadowing effects. In fact, under the selected deposition conditions, the effect of the angle is to increase the decoration depth along the NWs sidewalls, promoting a more uniform coverage as the tilt angle increases. In order to demonstrate such behavior and to investigate the effect of the oblique angle deposition on the NWs structural and optical properties, a set of fractal NWs samples having length of 2.5 µm were decorated at three different angles, 5°, 10° and 15°. For each angle, Er:Y_2_O_3_ films were simultaneously deposited onto Si bulk substrates to be used as references. The Er:Y_2_O_3_ stoichiometry was verified unchanged for all the deposited samples, as confirmed by Rutherford Backscattering Spectrometry (RBS) measurements discussed in the supplementary information S1. Indeed, for all the tilt angles elemental doses of Er, Y and O are in the ratio (Er + Y):O = 2:3 with about 4.5 × 10^17^ at/cm^2^ and 7 × 10^17^ at/cm^2^ for yttrium and oxygen, whereas a total dose of 0.3 × 10^17^ at/cm^2^ for erbium, corresponding to an Er concentration of about 2 ± 1 at%. All the NW samples and Si references were placed onto the same position of the tilt holder, in order to guarantee a good uniformity both in terms of morphological and optical response.

### Fractal characterization

Figure 1e shows a SEM plan view image of the bare NWs before the decoration process. As already demonstrated^12,35^, these Si NWs arrays are artificial 2D random fractals prepared through metal-assisted chemical etching by using percolative films of gold as a catalyst. Above the percolation limit the Au precursor layer has a fractal arrangement^36,37^ whose complementary disposition is transferred onto the Si NWs during the etching with a well controlled, maskless, low cost, and industrially compatible method. A fractal is realized by the recursive repetition of a structure along the space with scale invariance and self-similarities^38^. In particular, our NWs arrays are finite fractals displaying strong self-similarities over a wide range of length scales, from tens of nanometers up to a few microns. We have already demonstrated that the NWs fractal peculiar morphology leads to a strong light trapping across the whole visible and near-IR range due to the efficient in-plane multiple scattering within the NWs layer^12^, with promising potentialities for both photovoltaics and photonics applications^11^. The remarkable refractive index fluctuation across the NWs layer allows the enhancement of the optical response of the system as a function of the fractal arrangement^39,40^.

In this work, we obtained the decoration of the NWs fractal arrangement with Er:Y_2_O_3_ without destroying the fractal morphology. By varying the sputtering deposition angle θ from 5°, to 10° and 15°, we demonstrate the ability to tune the fractal morphology with an industrial compatible and cost-effective approach. The SEM plan view microscopies of bare and Er:Y_2_O_3_ decorated Si NWs at 15° are compared in Fig. 1e, f, respectively. By comparing the two images reported at the same magnification scale, it is possible to attest that the NWs random fractal arrangement is still preserved after the decoration process but on a different scale range, since the interstices among nanowires are now evenly covered with the Er:Y_2_O_3_ structures (see Supplementary Information Sect. 3). Although the fractal structure is still present, it has changed after the decoration and the bigger holes surrounding the NWs are slightly reduced in size.

The key parameters of the fractal, such as the filling factor, lacunarity, and fractal dimensions, were investigated for all samples by software analysis of the high resolution SEM plan view images, as described in the experimental section. As confirmed by a complete fractal analysis reported in Table 1, we demonstrated the realization of a random fractal structure of Er:Y_2_O_3_ decorated Si NWs, attesting that it is possible to change the fractal arrangements by varying the deposition angle of the substrate.

**Table 1:** Structural and fractal parameters of bare Si NWs and Si NWs samples decorated with Er: Y_2_O_3_ as measured from ImageJ software analyses of the SEM plan view images.

Name	Filling factor Si + Er:Y_2_O_3_	Filling factor Er:Y_2_O_3_	Fractal dimension D_F_	Lacunarity maximum (nm)
NW bare	42% ± 2%		1.88 ± 0.02	53 ± 1
5°	76% ± 1%	34% ± 1%	1.97 ± 0.01	120 ± 3
10°	74% ± 1%	32% ± 1%	1.96 ± 0.02	160 ± 6
15°	69% ± 1%	27% ± 1%	1.94 ± 0.02	190 ± 10

### Structural characterization

**Figure 2:**
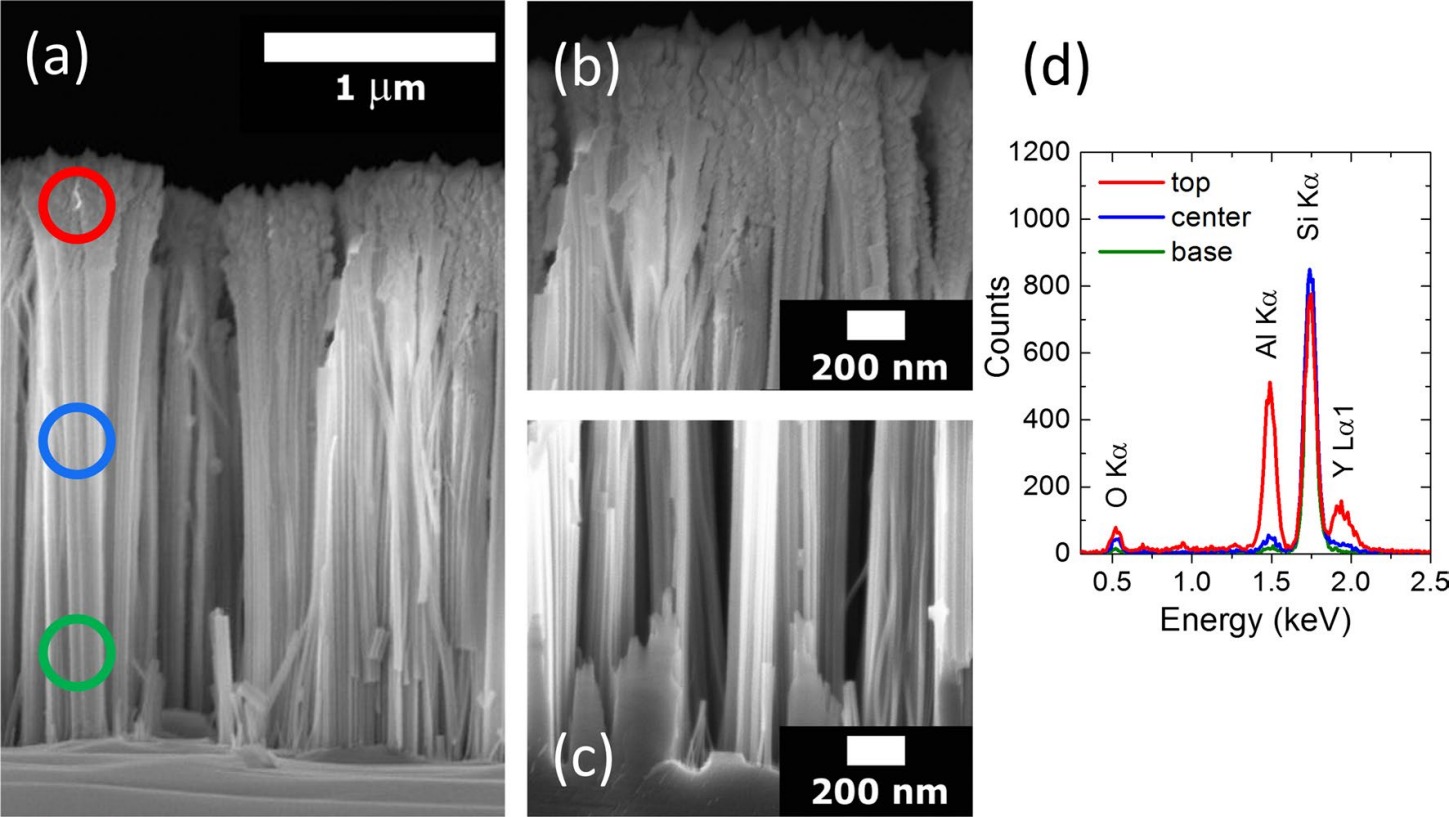
(**a**) SEM cross sections of Si NWs decorated with Er: Y_2_O_3_ at 10° by oblique angle deposition. The decorated NWs (**b**) tips and (**c**) bottom sections are shown at higher magnification, respectively. (**d**) EDX spectrum acquired onto top (red line), center (blue line), and bottom (green line) regions of the decorated NWs arrays highlighted with circles of respective colors as shown in (**a**).

The morphology of Er:Y_2_O_3_ decorated Si NWs was investigated by SEM both in plan view and in cross section per each tilt angle. It is worth noticing that the Si NWs arrays are not damaged during the decoration process and the presence of a uniform Er:Y_2_O_3_ coverage is appreciable from the NWs plan view for all the investigated samples. In order to evaluate the NWs sidewalls coverage, we have performed also SEM images in cross section. As a representative of the ensemble, Fig. 2a shows the SEM cross section of the sample decorated at an angle of 10°, viewing the whole decorated array, while Fig. 2b, c display the high magnification details of the same array corresponding to the top and bottom sections, respectively. The presence of a polycrystalline Y_2_O_3_ structure decorating the uppermost part of the NWs tips is clearly visible in Fig. 2b. While in Fig. 2c the Y_2_O_3_ presence is no longer recognizable along the NWs side wall as soon as we approach to the innermost section of the array. On the other hand, the oblique angle depositions on the flat Si bulk references resulted in continuous films of Er:Y_2_O_3_, with unchanged stoichiometry by increasing the tilt angle.

In order to investigate the dependence of the Er:Y_2_O_3_ distribution along the nanowires side walls on the tilt angle, we have performed energy dispersive X-ray (EDX) spectroscopy analysis. EDX spectra recorded along the NWs length in the top (red circle), central (blue circle) and bottom (green circle) sections have been reported in Fig. 2d for the same sample deposited at 10°. In all the three zones, the signals relative to Si from Si NWs and Y and O from the deposited matrix have been detected. Since the Er concentration in the samples is low, 2 at% uniformly dispersed in the Y_2_O_3_ matrix, its X-emission lines are not detectable by EDX analysis. In the central and base regions we attested a reduction of the Y Lα1 signal (1.9 keV) down to 16% and 4% of the value measured on the NWs tips.

The oxygen concentration behaves similarly, decreasing to the 54% and 20% of its value on the top for the Kα emission line of O at 0.5 keV, while the Si Kα emission line at 1.75 keV indicates that the Si concentration experiences a 5% decrement in the top region due to the major presence of Y_2_O_3_.The discrepancy in the Y and O percentage reduction can be explained by considering that the O estimation is also affected by the presence of a SiO_2_ shell surrounding the NWs. The EDX depth profiles evidenced similar concentration trends as a function of the position along the NWs sidewalls for all the investigated tilt angles. Though a precise estimation of the length covered by Er:Y_2_O_3_ cannot be given with high accuracy, we can assert that at a depth of about 1.5 µm from the top, the presence of Y_2_O_3_ is reduced below 10% of its concentration on the NWs tips for all the tilt angles. Moreover, we can exclude the formation of Er silicate at the interface between Er:Y_2_O_3_ and Si NWs due to the low substrate temperature of 300° C used during the deposition, as already demonstrated in literature^33,41^.

Indeed, the absence of Er-silicates in Er:Y_2_O_3_ decorated NWs was also confirmed by X-ray diffraction (XRD) analysis and no evidence of the erbium silicate characteristic peaks was found, as reported in fig. S2 of the supplementary info.

### Filling factor dependency from the deposition tilt angle

In order to demonstrate that with this oblique angle sputtering decoration it is possible to tune the fractal morphologies of Er:Y_2_O_3_/NWs, we carried out a detailed analysis on the critical fractal parameters.

The main visible effect of the decoration is an increment of the fill factor (FF), namely defined as the ratio of the covered area per the total area of the analyzed image measured by pixel counting (details are reported in the Supplementary Information Section S3). A FF of about 42 ± 2% was measured for bare Si NWs. After the deposition of an equal amount of Er:Y2O3, the decorated NWs samples at 5°, 10° and 15° angle show FFs of 76 ± 1%, 74 ± 1% and 69 ± 1%, respectively (as reported in Table 1).

These results on the fill factors highlight a strong correlation with the tilt angle. In fact, the FF value decreases as the tilted deposition angle increases, suggesting that a major amount of Y_2_O_3_ is distributed along the NWs sidewalls than onto their tips. In agreement with the expected shadowing effects, the decoration depth reached by the yttria along the vertical NWs profile increases for higher angles. This effect can be understood considering that for higher angle the fill factor decreases, thus decreasing the aperture of the shadowing cone. As a consequence, the morphology of Er:Y_2_O_3_ matrix can be simply varied in the x–y plane of the NWs due to the effect of the oblique angle deposition. Moreover, a typical feature of a fractal arising from their scale invariance is the measurement of the same surface coverage percentage for different magnifications. Indeed, the fractal array of Si NWs shows about the same fill factor for all the three investigated magnifications of 5kX (low), 50 kX (medium) and 300 kX (high).

Similarly to the fractal array of bare Si NWs, Er:Y_2_O_3_ decorated Si NWs present the same FF for the three low, medium and high magnifications measured in order to attest the fractal geometry of the decorated system. It is worth noticing that smaller holes are no longer appreciable for higher magnification (above 300 kX) due to the decoration coverage.

### Fractal parameters correlation

Such self-similarity attested by the fill factor is typical of fractal geometries, which present scale invariance extended over a wide length scale^42^. Thus, in order to investigate the fractal nature of Er:Y_2_O_3_ decorated Si NWs arrays, the fractal characteristic parameters of fractal dimension (D_F_) and lacunarity (L_c_ ) were measured as a function of the scale invariance of the system. The fractal dimension measures the complexity of the structure, attesting how the pattern scales as a function of the investigated length-scale^35^. The methodology used and the fractal parameter calculation procedures are described in details in the Supplementary Information Section S3.

As expected, we calculated a D_F_ of about 1.88 ± 0.02 for bare Si NWs, corroborating the claim that these Si NWs arrays display a 2D random fractal arrangement, as previously demonstrated in details^12^. Similarly, Er:Y_2_O_3_ decorated Si NWs behave as statistical fractals, presenting a non-integer D_F_ value varying slightly from 1.97 to 1.94 when the deposition tilt angle increases. Nonetheless, D_F_ does not describe univocally a fractal pattern and it may occur that two different fractal arrangements scale with the same fractal dimension. Indeed, the main parameter which univocally describes the structural properties of a fractal is the lacunarity (L_c_, mathematically defined according to the method shown in details in Supplementary Information Section S3). Lacunarity measurements are used to uncover scale-dependent changes of a spatial structure, since it is related to the deviation of fractals from translational invariance. It provides complementary information to the fractal dimension, describing how the fractal texture is organized in terms of the fluctuation of the distribution of the alternation of empty and filled spaces, i.e. heterogeneities^43^. The lacunarity is related to the variance of the hole size distribution measured at different magnification scales, probing the repetition of the same morphology typical of fractals. Indeed, those fractals whose heterogeneities present high fluctuations lead to a high lacunarity for the investigated range. In particular, random and dense fractals are expected to show high lacunarity (L_c_ > 1). Meanwhile, totally homogeneous structures show no lacunarity (L_c_ = 1). The trend of the lacunarity was investigated per each sample as a function of the length scale, and the length value at which the lacunarity assumes its maximum are resumed for all samples in Table 1. The length scale at which the lacunarity peak is measured corresponds to the maximum fluctuation of heterogeneities of the system, determining a strong fluctuation of the refractive index and thus increasing the scattering strength of the material, as well known in literature^12,36,44^.

Bare Si NWs present significant heterogeneity fluctuations across dimension range from 30 nm up to 200 nm. Their lacunarity peak reaches the maximum value of 1.35 at the scale of 53 nm and a full width at half maximum (ΔL_c_) of about 50 nm. Therefore, 2D random fractal array of bare Si NWs presents strong heterogeneity fluctuations at smaller length scale of about 50 nm. It can be concluded that the hole size distribution in bare Si NWs show huge fluctuations for dimensions below 200 nm.

As expected, by using oblique angle sputtering decoration it is possible to modify the lacunarity of the system. The intensity of the lacunarity peak decreases of about the 51%, 43% and 40% with respect to bare NWs, shifting towards bigger length scale of 120, 160 and 190 nm for the 5°, 10°and 15° decorated NWs samples, respectively. The deposition tilt angle is a key parameter that affects both the fill factor and lacunarity, determining how the material is distributed onto the fractal NWs structure. In the end, higher lacunarity and smaller fill factor values are measured by increasing the deposition angle. Indeed, for higher deposition angle the Er:Y_2_O_3_ decorate a deeper section along the NWs vertical profile decreasing the fill factor and thus increasing the density of bigger holes of random size, while smaller holes among NWs are occluded with the deposited material. Since the smallest holes are occluded, the fluctuation of the holes size distribution decreases at a smaller scale thus shifting the lacunarity peak toward a higher length region. On the other hand, the density of larger holes is now dominant and the fluctuation of its statistic distribution increases toward a higher and broader scale. Moreover, the extension of the heterogeneity fluctuation range (ΔL_c_) also increases due to the shadowing effect when the decoration angle increases since bigger holes remain partially uncovered with a size distribution that is highly variable over a broader region (up to 800 nm). It can be concluded that the morphology and structural parameters of 2D random fractal arrays of decorated Er:Y_2_O_3_ Si NWs can be controlled by varying the sputtering deposition angle. In fact, the most disordered lacunarity conditions is achieved at an angle of 15°, where a maximum value of 1.18 is observed for the lacunarity peak, which extends over a broader region with ΔL_c_ ≈ 340 nm.

### Photoluminescence properties

**Figure 3:**
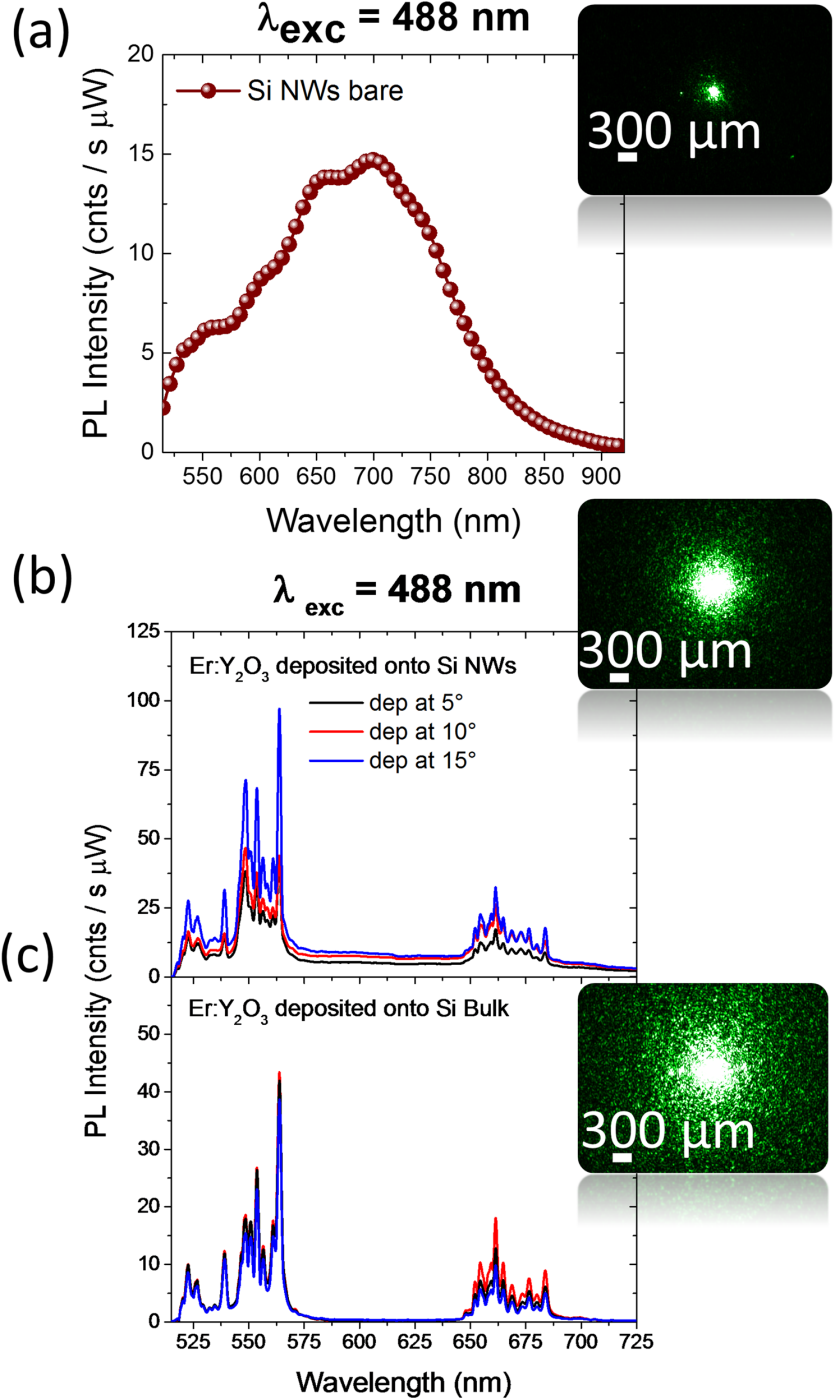
(**a**) Visible PL emission at about 700 nm observed for bare Si NWs before the decoration at the excitation wavelength of 488 nm. (**b**) PL spectra of Er:Y_2_O_3_ decorated Si NWs samples reported as a function of the deposition angle and compared to their (**c**) Si bulk references at the same excitation wavelength of 488 nm. The insets shows the optical microscopy investigation of the laser pump beam scattered out of plane for the three presented substrates: (**a**) Si NWs bare, Er:Y_2_O_3_ decorated (**b**) Si NWs and (**c**) Si bulk deposited at the same conditions.

The characterization of the optical response of the bare NWs, Er:Y_2_O_3_ decorated NWs and Er:Y_2_O_3_ films onto Si bulk substrate have been tested by photoluminescence (PL) spectroscopy (Fig. 3).

The PL spectrum of bare Si NWs acquired at the excitation wavelength of 488 nm is reported in Fig. 3a. NWs typical PL emission band is centered at about 700 nm due to quantum confinement effect^35^ . We observed the different scattering of the decorated NWs system by bright-field microscopy (BF) acquired under laser illumination, as shown in the inset of Fig. 3a. The BF microscopy of bare Si NWs shows that most of the light is trapped inside the NWs fractal due to their strong scattering, caused by the modulation of the refractive index of the system^12,35^. The Er visible photoluminescence from Er:Y_2_O_3_ onto Si bulk and onto NWs were also tested as a function of the deposition tilt angle under excitation wavelength of 488 nm, resonant with the ^4^I_15/2_ → ^4^F_7/2_ Er^3+^ transition, as reported in Fig. 3b,c, respectively. All the PL spectra were obtained at room temperature, under the same experimental conditions, normalized to the pump power, and averaged on different points to account for the uniformity of the system.

The PL spectra from all the Er:Y_2_O_3_ show the characteristic Er emission from the multiplets ^4^S_3/2_ and ^4^F_9/2_, peaked around 560 and 660 nm respectively. However, different PL dependence on the deposition angle are observed for the films onto Si with respect to the decorated NWs. As expected owing to the unchanged Er content and distribution, no relevant variation is observed as a function of the deposition angle in the Er:Y_2_O_3_ films onto Si bulk (Fig. 3c). On the contrary, the Er PL intensity from Er:Y_2_O_3_ decorated NWs increases by increasing the tilt deposition angle (Fig. 3b), suggesting the dependence of the optical properties on the fractal morphology. We measured a PL increment for the Er band at 560 nm of a factor of 1.1 for the 10° NWs sample and 2.4 for the 15° one with respect to the sample deposited at 5°. Moreover, the Si NWs luminescence band is no longer observed since it is less efficient than Er emission.

This peculiar behavior suggest that the deposition angle θ affects the optical emission, since it determines the fractal morphology and in particular, its lacunarity. Indeed, the different lacunarity determine the refractive index modulation, leading to a strong scattering inside the material. This effect allows to enhance the excitation of the system, hence the Er emission intensity^36,44,45^.

Thus, the observed Er PL improvement in the decorated Si NWs can be ascribed to the enhancement of the excitation pump through scattering effects driven by the fractal parameters^12,46,47^.

The bright-field microscopies were acquired under the same experimental conditions also for Er:Y_2_O_3_ deposited at the same angle of 15° onto Si NWs and Si bulk, as reported in the inset of Fig. 3b, c, respectively. From the comparison among all the samples it can be observed that the extension of the scattered light increases when passing from bare Si NWs to Er decorated ones. Figure 3c shows that most of the light is indeed scattered out in the Er:Y_2_O_3_ decorated Si bulk samples, suggesting that the light is better trapped into the Er:Y_2_O_3_ decorated NWs.

**Figure 4:**
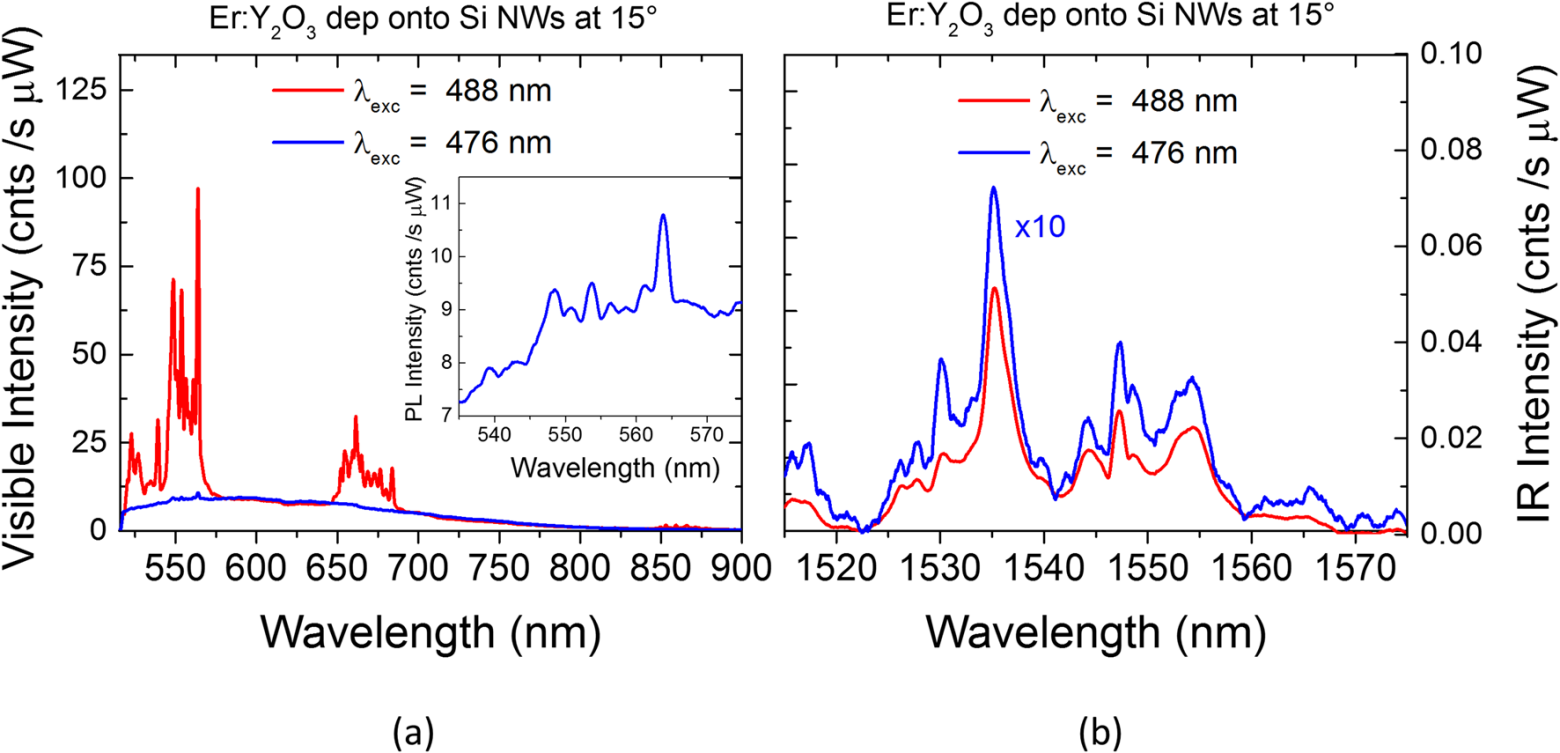
PL spectra of Er:Y_2_O_3_ decorated Si NWs at 15° obtained at the excitation wavelengths of 488 nm (red line) and 476 nm (blue line) are shown for both (**a**) visible and (**b**) infrared range. The inset in (**a**) shows the Er emission band at 560 nm for the indirect excitation wavelength of 476 nm.

In order to evaluate an eventual role of mediated excitation from Si NWs to Er ions, Erbium PL spectra for decorated Si NWs at 15° are shown in Fig. 4 over an extended range, from the visible to the infrared regions, under both direct (488 nm) and indirect (476 nm) excitation reported in red and blue, respectively. Under indirect excitation, the Er PL spectra are very low in intensities for both visible and infrared emission, corresponding to the de-excitation from the ^4^S_3/2_, ^4^F_9/2_, and ^4^I_13/2_. The observed broad emission band at about 600 nm can be mainly attributed to eventual defects, and is also present as a background for the direct excitation (red spectrum). Therefore, we can conclude that no energy transfer occurs under both direct and indirect optical pumping. However, a PL increment for the decorated NWs with respect to Si bulk is observed for both visible and infrared emission for all the excitation wavelengths and tilt angles (all the spectra are reported in Fig. S4 of the Supplementaty information Sect. 4). Hence, the observed emission increment for the visible and infrared bands could be ascribed to the scattering enhancement in the NWs samples, since all the emission bands increases and not only the resonant ones.

**Figure 5:**
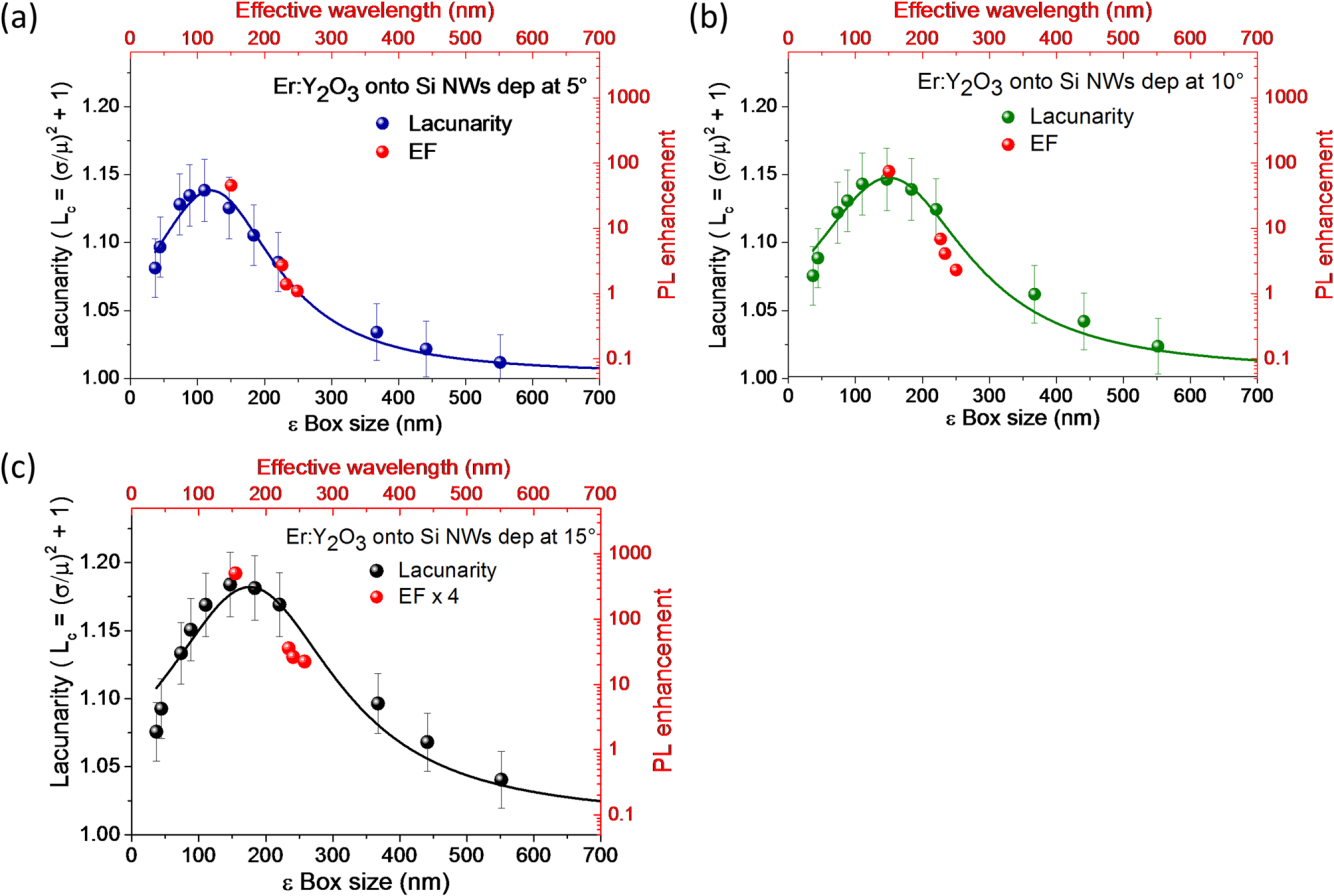
The normalized PL enhancement factors (EF) per probed volume calculated for the Er emission band at 560 nm (red dots) are compared to the lacunarity of Er:Y_2_O_3_ decorated Si NWs sample at 5° (**a**), 10° (**b**), 15° (**c**). The EF of the 15°decorated sample is reported multiplied for 4 in order to use the same arbitrary scale of PL enhancement for all the samples.

The Er emission increment on NWs suggests that optical response is improved by the fractal structure tuned by the tilt angle. To correlate the optical response of the system to the lacunarity of the fractals, we compared their lacunarity to their respective Er PL enhancements, as reported in Fig. 5. The photoluminescence of the Er:Y_2_O_3_ deposited onto Si NWs and Si bulk were measured by using the following excitation wavelengths of 514 nm, 488 nm, 476 nm and 364 nm, and all the spectra were normalized for the acquisition for the comparison. For each deposition, the PL enhancement factors has been defined as the ratio of the integrated intensity for Er 560 nm emission band measured onto Si NWs (I_NWs_) with respect to the film on Si bulk (I_film_) and normalized for a scaling factor (f_Er_) accounting for different amount of Er emitting centers effectively excitable per each excitation wavelength.

As far as the Er film deposition on Si bulk is concerned, all the Er emitting centers are distributed uniformly along a thickness of about 160 nm for the deposition on flat Si and thus are all optically excitable for the studied excitation wavelengths range. Instead, the Er concentration along the Er:Y_2_O_3_ decorated Si NWs decreases from their tip to the bottom up to 1.5 µm for 15° (as well attested by the EDX analyses), thus we considered the effective fraction of Er ions that can be probed by the laser illumination.

Indeed, our structural investigation (reported in Table 1) confirms that the Er:Y_2_O_3_ decorated NWs are mainly made of a 42% of Si and a percentage of Y_2_O_3_ matrix (doped with 2% of Er) varying from 34 to 27% according to the deposition angle from 5° to 15°, consequently the remaining percentage will be composed of air (from about 24 to 31%). Since the Y_2_O_3_ matrix is highly transparent from 300 nm up to the whole visible region, its absorption within the decorated fractal system can be neglected. The absorption of the 42% composition of Si must be considered especially in the UV region, where the light penetration depth (δ_p_) is about 27 nm for the 364 nm excitation. Conversely, for the 476 nm, 488 nm and 514 nm excitations the light penetration depths are considerably higher, corresponding to 1050 nm, 1190 nm and 1665 nm, respectively.

We can estimate that the Er profile is extended up to 1.10 µm, 1.25 µm, and 1.5 µm for deposition at 5°, 10° and 15°, respectively, considering unchanged the total Er content (as confirmed by RBS) and the increased FF values (see Table 1). Thus, for all the deposition angles, we can assume that all Er ions will be optically excitable at the wavelength of 514 nm. Instead, at the wavelengths of 488, 476 and 364 nm the light penetration depth is lower than the Er ions extension, and the effective fraction of emitting centers involved has been evaluated keeping constant the total Er content. Therefore, we have estimated the PL enhancement factor as the ratio of erbium 560 nm PL band measured on Si NWs (I_NWs_) with respect to bulk (I_film_) and normalized for the fraction of optically active centers for each excitation wavelength, as described in the following equation:

1 $$EF=\frac{{I}_{NWs}}{{I}_{film}} {f}_{Er}$$

where *f*_Er_ = L_Er_ / δ_p_ with δ_p_ penetration depth of the light and L_Er_ the Er profile extension of the decorated Si NWs sample.

For the all cases where the light penetration is higher than the maximum Er profile extension we considered *f*_Er_ = 1. A more detailed discussion on the scaling factors is reported in the supplementary information S4.

The PL enhancement factors for the decoration at 5°, 10°and 15° are reported as red dots and plotted as a function of the effective incident laser wavelength propagating into the medium λ_eff_ (red top axis) in Fig. 5a–c, respectively. For each sample, the λ_eff_ was obtained by the Bruggeman mixing rule, assuming that all samples have the same composition of 30% of Si and 15% of SiO_2_ (measured from bare Si NWs) and different percentages of air and Y_2_O_3_ measured from the fill factor and confirmed by EDX analyses (see Supplementary information Sect. 4, Table S1).

The PL enhancement trends were compared to the lacunarity of each sample, as shown in Fig. 5a–c. A good match between the trends of the PL enhancement and the lacunarity of the systems are reported for all the investigated samples. Indeed, the lacunarity peak determines the conditions at which the excitation wavelength is better scattered inside the system, thus increasing the excitation pumping.

The effective wavelength range (top red axis ) is compared to the length scale of the lacunarity (bottom black axis) in Fig. 5. The effective wavelength ( λ_eff_ = λ/*n*) is slightly different for each sample since *n* depends on the filling factors (Bruggeman mixing rule), while the lacunarity depends on the fluctuation of the pixel density (refractive index fluctuation) at different length scale, and not only from the filling factors. Hence, small EF shifts are not relevant, what is expected is that the relative EF modulation follows the same variation of the lacunarity. Indeed, the maximum refractive index fluctuation corresponds to lacunarity peak, at which the multiple scattering across the structure is maximized, as previously shown for not decorated Si NW material^12^. As a consequence, the scattering increases the Er excitation, and the EF will behave in the same way of the lacunarity, as attested in Fig. 5.

These results suggest that the fractal morphology, controlled by the oblique sputtering deposition, leads the increment of Er emission in Si NWs fractal systems.

## Conclusions

We demonstrated the capability to realize novel materials onto Si NWs with fractal morphology whose structural arrangements can be controlled with a low cost and industrial approach. Indeed, we demonstrated that the fractal structure of Si NWs arrays is modified with Er:Y_2_O_3_ decoration by oblique sputtering. When in presence of the fractal NWs, Er photoluminescence shows a PL enhancement for its 560 nm band that matches the morphology of the system, thus corresponding to the effective wavelength range at which the fluctuation of the refractive index is maximized. This result opens new perspectives in the field of light management in novel Si-compatible photonic materials.

## Material and methods

### Materials

n-doped (ρ ≈ 1–5 Ω∙cm) Si wafers of 4″ were purchased from Siegert Wafer GmbH, 50% Hydrofluoridric Acid (HF) was purchased by Sigma-Aldrich, 4 N Au pellets of 1/8″ were acquired by Cinque Pascal and deposited onto Si substrate for the NWs synthesis by electron beam evaporation. AJA sputtering targets of Er_2_O_3_ and Y_2_O_3_ with 99.99% purity were used.

### Silicon nanowires synthesis

Si wafer were cut into 1 × 0.5 cm^2^, threated with UV cleaning for 2 min and rinsed in HF 5% solution for 5 min to obtain an oxide-free clean surface. Silicon nanowires were synthesized from the chemical etching of Si substrates in a HF (5 M) H_2_O_2_ (0.4 M) aqueous solution assisted by the presence of thin metal film deposited by electron beam evaporation at room temperature with a KS 500 Kenosistec evaporator. When in solution, Si oxidation is catalyzed by the metal film, leading to the local formation of SiO_2_ underneath the metal covered region that is selectively etched by the HF. During the etching, the Au film sinks into the Si bulk leading to the formation of a dense forest of Si NWs. Each step of the NWs formation is performed at room temperature, preventing any gold contamination into the Si NWs. The Au thin layer is removed afterwards by a selective gold etchant solution from Sigma Aldrich.

### Er:Y_2_O_3_ decoration of Si NWs

Silicon nanowires were decorated with erbium doped yttrium oxide grown by ultra-high vacuum magnetron co-sputtering performed at different oblique angles (5°, 10°, 15°). The Y_2_O_3_ and Er_2_O_3_ targets were eroded by radiofrequency co-sputtering in a controlled Ar atmosphere at a pressure of 5 × 10^–3^ mbar and deposited onto Si NWs and c-Si (111) reference substrates placed onto the uppermost part of a tilted homemade holder with an area of about 8 cm^2^, heated at a constant temperature of 300 °C. A fixed amount of yttrium oxide and erbium was used for all the decorations, maintaining constant powers applied to the Er_2_O_3_ and Y_2_O_3_ targets (35 W and 500 W, respectively) and constant deposition pressure of 5.0 × 10^–3^ mbar.

### Characterization

The scanning electron microscopy (SEM) images and energy dispersive X-ray analysis (EDX) were performed using with a ZEISS Field Emission microscope (SUPRA) equipped with an InLens and energy-dispersive X-ray detectors (EDAX), respectively. The films thickness was measured by SEM analysis performed on the Si bulk reference substrates and confirmed by Rutherford Backscattering Spectrometry (RBS).The elemental composition of Er:Y_2_O_3_ decorated Si and Si NWs samples were measured by RBS, performed by colliding a He ^+^ beam at an energy of 2 MeV onto the samples. The backreflected He^+^ ions were collected at the detection angle of 165° with respect to the beam direction and their energy loss has been investigated by a multichannel analyzer.

The fractal parameters of lacunarity and fractal dimension were measured by using the non-overlapping box counting algorithm implemented in the FracLac plugins of ImageJ analysis software. SEM microscopy with magnification of 50 kX were sectioned in grid of variable dimension and the pixel density was measured to calculate both fractal dimension and lacunarity. Photoluminescence spectra were acquired by using a HR800 Lab spectrometer from Jobin–Yvon Horiba equipped with cooled CCD and InGaAs array detectors for visible and infrared spectroscopy, respectively. The samples were pumped at the excitation wavelengths of 476, 488 and 514 nm lines obtained from an Ar^+^ laser focused onto the sample by means of a 100× objective (NA 0.9) with a power of about 30 µW measured onto the sample plane. The bright-field scattering microscopies of the samples were acquired by focusing the 514 laser line at a power of 100 µW onto the sample through a 100× objective and collected through the same objective with a uEye microscopy camera.

## Supplementary information

Supplementary file1 (DOCX 12883 kb)
