# Supplementary material for: Erbium emission in Er:Y2O3 decorated fractal arrays of silicon nanowires
Source: Sci Rep. 2020 Jul 30;10:12854. doi: 10.1038/s41598-020-69864-5 (PMC7393374; doi:10.1038/s41598-020-69864-5)
Supplement: Supplementary file 1 — Supplementary file1 (DOCX 12883 kb) [file 41598_2020_69864_MOESM1_ESM.docx]

**Supplementary Information**

Erbium emission in Er:Y_2_O_3_ decorated fractal arrays of silicon nanowires

M.J. Lo Faro^1,2,3^, A.A. Leonardi^1,3,2^, F. Priolo^1,2^, B. Fazio^3^, M. Miritello^2*^, A. Irrera^3*^

^1^ Dipartimento di Fisica e Astronomia “Ettore Majorana”, Università di Catania, Via Santa Sofia 64, 95123 Catania, Italy.

^2^ CNR-IMM, Istituto per la Microelettronica e Microsistemi, Via Santa Sofia 64, 95123 Catania, Italy.

^3^ CNR-IPCF, Istituto per i Processi Chimico-Fisici, V.le F. Stagno D’Alcontres 37, 98158 Messina, Italy.

* corresponding authors: M. Miritello ([maria.miritello@ct.infn.it](mailto:maria.miritello@ct.infn.it)), A. Irrera ([alessia.irrera@cnr.it](mailto:alessia.irrera@cnr.it))

**1. Rutherford backscattering spectroscopy (RBS) measurements**

The elemental composition of Si NWs decorated with Er-doped Y_2_O_3_ matrix deposited by oblique angle magnetron sputtering was investigated by Rutherford Backscattering Spectrometry (RBS). RBS is a technique that allows the measurements of the surface atomic density (at/cm^2^) of elements heavier than the probing beam (He^+^) whose energy loss spectrum is collected after the interaction with the target of interest. The RBS measurements were performed by using a singletron HVEE accelerator for He^+^ ions and the backscattered ions were collected and analyzed with a multichannel analyzer. The RBS spectra measured for the Si bulk samples after the Er: Y_2_O_3_ depositions are very similar for all the three investigated angles of 5°, 10° and 15° and the same signals relative to the atomic presence of O, Si, Y and Er are evident. For clarity, in fig. S1 we reported only the RBS spectra measured on Si NWs before the deposition (shown in green) and after the Er:Y_2_O_3_ decoration at the angle of 10° onto Si bulk and Si NWs represented by the red and blue spectra, respectively. The RBS spectra of Si bulk were simulated for each deposition angle by using the SIMNRA simulation suite to estimate the elemental composition of the films, confirming that all the deposited samples show the same Er:Y_2_O_3_ stoichiometry. As expected, no variation of Er:Y_2_O_3_ atomic density is observed and from the peak integration, an Er concentration of 2 ± 1 at% (areal density of about 0.3×10^17^ at/cm^2^) has been estimated, while Y and O show concentrations of about 38 ± 2 at% (areal density of 4.5×10^17^ at/cm^2^) and 60 ± 5 at% (areal density of 7×10^17^ at/cm^2^), respectively. RBS is not a suitable measurement for nanostructures since it is sensitive to the presence of voids and cavities that strongly affects the energy loss profile across the material. Thus, the RBS measurements performed onto the bare NWs present a smooth decreasing shoulder for the He^+^ ions energy loss instead of the expected sharp profile attested on Si bulk, making the comparison more complex due to the presence of the surface roughness. Nonetheless after the Er:Y_2_O_3_ deposition, the Si signal shows a clear shift towards lower energy loss channels which is the results of the addition energy loss of the ion probe that is proportional to the Er:Y_2_O_3_ concentration onto the surface. It is worth noticing that after the Er:Y_2_O_3_ decoration, both the decorated Si bulk and Si NWs attest about the same downshift, further suggesting the same concentration of Er, Y and O deposited on the two substrates. This point is
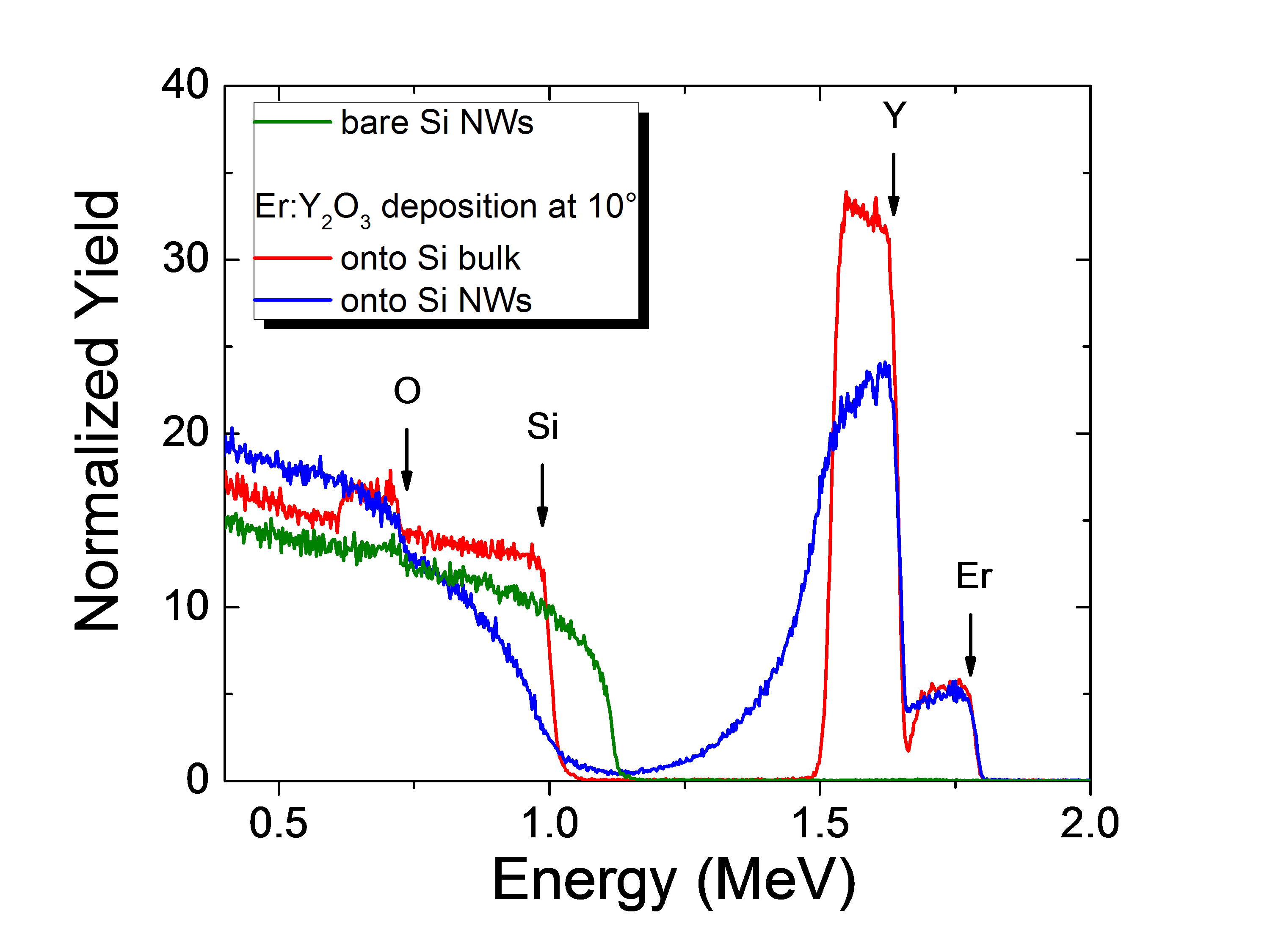
strongly confirmed by the same Er and Y areal densities in the two samples.

Figure S.1 : **Compositional analysis:** Rutherford backscattering measurements performed on the Er:Y_2_O_3_ deposition on Si bulk and on Si NWs realized at the sputtering angle of 15°. The green spectrum correspond to the signal acquired from bare Si NWs.

**2. X-Ray Diffraction (XRD) measurements**


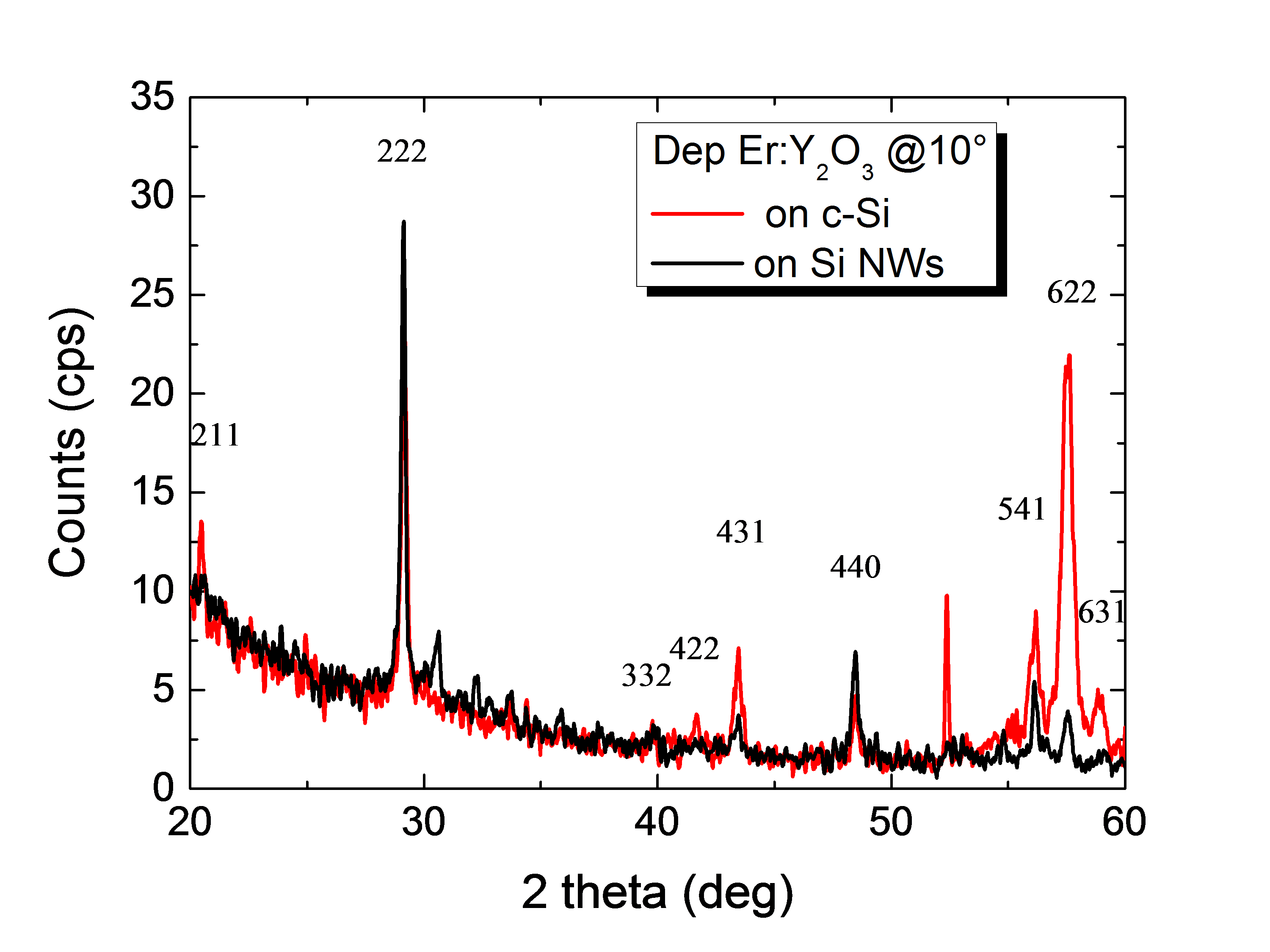
As well known from literature, the interaction of Er oxide with a silicon substrate may result in the formation of Er silicates at the interface. Therefore, we investigated the presence of Er silicates by X-ray diffraction (XRD) analyses performed onto Si bulk and Si NWs by using a Bruker X-ray Diffractometer. The good crystalline quality of the Er:Y_2_O_3_ films is confirmed for both the samples deposited onto Si bulk (red spectrum) and onto Si NWs (black spectrum) at the same angle (10°), as attested by the XRD spectra reported in fig. S2. The comparison of the XRD spectra shows the identical fingerprint of the cubic Y_2_O_3_ crystalline structures for the two samples, confirming the absence of silicates whose characteristic X-ray diffraction peaks are not visible.

Figure S2. **Crystalline structure:** X-ray Diffraction spectra obtained in the 2 theta configuration for a deposition of Er:Y_2_O_3_ film onto Si bulk (red spectrum) and onto Si NWs (in black) at the sputtering angle of 10°.

**3. Calculation of the Fractal parameters**

Finite fractal systems are characterized by the repetition of the same pattern across a defined set of length scale. To assess the fractal character of our Si NWs a detailed investigation of their morphologies was conducted by plan view scanning electron microscopies collected at different magnifications. As a representative example, the SEM plan view of fractal Si NWs before the decoration with Er is reported in fig. S3 at the magnifications of (a) 5kx, (b) 50kx, (c) 500kx. Such fractal NWs possess a random geometry that is recursively iterated with scale invariance, as attested by the SEM characterization. The fractal dimension and the lacunarity are the key parameters of a fractal, both related to the scaling of the recursive pattern and to the fluctuation of the filled pixel density probed as a function of the length scale. These fractal parameters were investigated for all samples by using the FracLac plugin of ImageJ software. FracLac sliding box counting algorithm works on binary images, thus the plan view SEM microscopies were first converted into binary images by ImageJ software. The same set of box sizes has been used for all the investigated samples in order to probe the translational invariance on the same scale-dimension range, and the same contrast threshold level was also used for consistency of the results. The 50 kX magnification scale was chosen since it allows to study a broad range from 20 nm up to 2 μm, simultaneously probing both big and small box dimensions, respectively well above and below hundreds of nanometers. Fig. S3 d-f show how the parameters were measured from the plan view of Si NWs decorated at 15° (fig. S3 e): (i) a grid (shown in magenta) was superimposed onto the SEM image and the number of filled pixel (in black) was counted (fig. S3 f). (ii) The process was iterated by varying the grid size from 37 nm up to 2.2 μm to achieve convergence, then (iii) repeated for five different initial grid positions in order to acquire a significant statistical ensemble. Three SEM images were investigated per each sample, scanning a total area of approximately 7.6×5 µm^2^ (1023×667 pixel^2^) to account for the uniformity of the deposition.

The number of boxes N mapping the structure (pixels occupied by filled spaces) was measured as a function of the box caliber *ε* (nm) by using the Fraclac ImageJ plugin. Afterwards, the number of occupied mean pixels per box N is plotted as a function of the investigated box sizes *ε* in a log-log scale and a linear relation is obtained. The fractal dimension (D_F_) is obtained from the slope of the linear function fitting the pixel density statistic per box size (as reported in the table 2 of the paper). This dimensionless measurements has nothing to do with the Euclidean dimension of the considered system and is generally defined as a measure of the complexity of the iteration of the fractal patterns. Fractal structures characterized by a non-integer D_F_ value below the Euclidean dimension of 2 are classified as two-dimensional random fractals. Indeed, fractal arrays of Si NWs have a DF of about 1.88 typical of 2D dense and random fractals. The fractal dimension measured after the decoration attest that also Er:Y_2_O_3_ decorated Si NWs are fractals with a characteristic D_F_ value from 1.97 to 1.94 when the deposition tilt angle increases. It can occurs that fractals with the same fractal dimensions have different fractal arrangements. Indeed, it is the lacunarity (L_c_) wich defines the morphology of the fractal system from the fluctuation of the pixel distribution σ^2^ per occupied box nomalized to the square of the average occupation value μ^2^ for each set of dimensions ε_i_:

L_c_ = 1+ σ^2^/μ^2^

where the pixel density distribution has been calculated by Fraclac plugins for a defined set of box size. In particular, the number of pixels describing the structure (black pixels shown in figure S3 f) is counted during the scan for each box in a grid placed on the image (shown in magenta). The correctness of the results was corroborated by calculating the lacunarity by means of a different sampling approach, measuring the number of pixels per grid by the overlapping sliding box counting. Similar results were obtained by both non-overlapping and overlapping box counting analysis, confirming the accuracy of the methods. Fig. S3 d shows the lacunarity trend for bare Si NWs (black spectrum), Er:Y_2_O_3_ decorated NWs at the angle of 5° (green), 10° (blue) and 15° (red) are also reported. The lacunarity trends were fitted with a Lorentian function and the lacunarity peak intensity (I_l_), position (l_x_) and full width at half maximum (ΔL_c_) were measured for each sample. The first parameter quantifies the fluctuation amplitude, the second indicates the length scale (grid dimension ε) at which the maximum fluctuation of heterogeneities is observed and is correlated to the structural resonances of the system. The latter one instead, measures the extension of the dimension range where the heterogeneities fluctuations are strongly present.

From the comparison of the lacunarity trends it can be observed that Si NWs present a sharp lacunarity peak with intensity of 1.35 centered at the length scale of 53 ± 1 nm whose intensity decreases significantly above 200 nm. It can be understood considering that the pixel density fluctuation is maximized for smaller length scale, while the hole sizes are evenly distributed with respect to the average value at higher range. From the comparison in fig. S3 e, it can be observed that lacunarity trends of the fractal NWs after Er:Y_2_O_3_ decoration varies as a function of the sputtering angle. Indeed, by increasing the sputtering angle from 5° (green spectrum) up to 15° (red spectrum) the lacunarity increases both in intensity and broadening, thus maximizing the heterogeneity of the system for higher deposition angles.


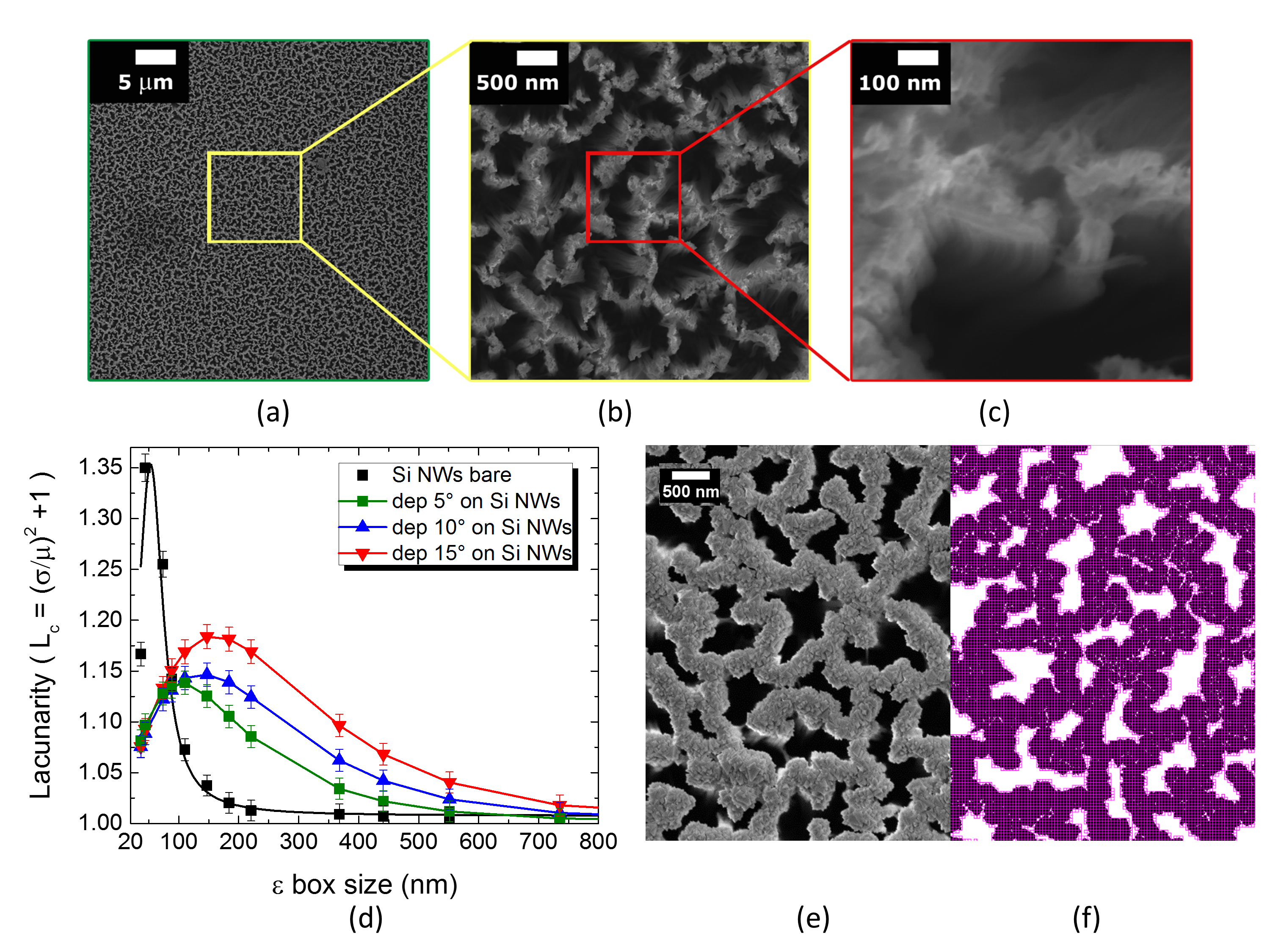
Indeed, this lacunarity behaviour is in good agreement with the achieved results on the filling factors, providing a strong confirmation that holes smaller than 100 nm are occluded with the deposited materials thus suppressing the hole size fluctuations observed for bare NWs at smaller length scales. For higher deposition angle, the material decorate a deeper section along the NWs profile resulting in a huge number of larger holes whose size vary randomly, increasing the fluctuation of the hole size distribution at higher length scale and on a broader region. As a result, the extension of the heterogeneity fluctuation range (ΔL_c_ ) increases while its fluctuation maximum is shifted towards higher values due to the decoration effect. Meanwhile, bigger holes remain partially uncovered and their size distribution is highly fluctuant over a broader region (up to 800 nm). It can be concluded that the morphology and structural parameters of 2D random fractal arrays of decorated Er:Y_2_O_3_ Si NWs can be controlled by varying the deposition angle affecting the refractive index fluctuation and the scattering of the system.

Figure S3: **Fractal characterizations:** (a-c) SEM plan view microscopies of bare Si NWs reported at three different magnification to test the scale invariance of the fractal structure. (d) Lacunarity of bare Si NWs before (black spectrum) and after the decoration at 5° (in green), 10° (in blue) and 15° (in red) from the fractal analyses performed on SEM plan view as shown in figure (e) and (f) after the pixel counting acquired for the magenta networks at scaling dimension.

**4. Optical properties**

In Fig. S4 are reported the PL spectra for both Er:Y_2_O_3_ decorated Si NWs and Si flat samples for all the used the excitation wavelengths. For all the PL measurements performed on decorated Si NWs and Si bulk we considered the integrated peak intensities subtracting the background at 560 nm fitted in a broader wavelength region (500-600 nm) than the integrated one (535-580 nm).





Figure S4: **PL measurements comparison for Er:Y_2_O_3_ decorated Si NWs and Si flat samples.** Erbium emission in the visible range reported for the deposition onto Si NWs (blue spectra) and onto Si flat (red spectra) and compared for all the used excitation wavelengths. All the measurements were normalized for the optical power measured onto the sample plane.

The effective wavelength propagating within the decorated array was calculated for each sample by using the Bruggeman mixing rule for the refractive index (*n*) evaluation starting from the composition of bare Si NWs made of Si and and SiO_2_ . The Si and SiO_2_ concentrations of 28% and 14 % respectively, were measured by energy filtered transmission electron microscopy (EFTEM) and energy dispersion X-ray spectroscopy (EDX) measurements^1,2^. The refractive index of the single components reported in the database from refractive index.info ^3^, for Si^4^, SiO_2_^5^, and Y_2_O_3_^6^_._

| **λ_eff_ = λ / *n*** | | |
| --- | --- | --- |
| **5°** | **10°** | **15°** |
| 150 ± 4 nm | 150 ± 4 nm | 155 ± 5 nm |
| 226 ± 6 nm | 227 ± 6 nm | 234 ± 7 nm |
| 232 ± 7 nm | 234 ± 7 nm | 241 ± 7 nm |
| 249 ± 8 nm | 250 ± 8 nm | 258 ± 8 nm |

Table S1: **Effective wavelength.** Resuming table of the effective wavelength calculated by the Bruggeman mixing rule for each decorated Si NWs samples.

In fig. 5 of the main text, we compared the lacunarity trend to the Er 560 nm emission band in order to demonstrate that the PL emission of the material can be increased by controlling its fractal structure and lacunarity. Indeed, a comprehensive explanation of the lacunarity calculation has already been provided, while a more detailed clarification for the calculation of the PL enhancement is discussed below. The PL enhancement could be easily calculated from the ratio of the PL intensity measured from Er deposited onto Si NWs (I_NWs_) with respect to Si bulk (I_film_), assuming that all the Er emitting centers are optically active and fully illuminated by the laser probe. Indeed, Er is uniformly distributed into the Y_2_O_3_ flat film deposited onto Si bulk over a depth of 160 nm, hence all the Er emitting centers are always illuminated in such condition due to Y_2_O_3_ transparency.

On the other hand, in the case of Er:Y_2_O_3_ decoration of Si NWs we need to consider that Er concentration decreases along their vertical profile (as well attested by the previous structural analyses). Hence, to calculate the enhancement factor (EF) we need to consider the portion of Er emitting centers effectively excited at the different wavelengths onto Si NWs with respect to the total amount, as sketched in fig. S5.


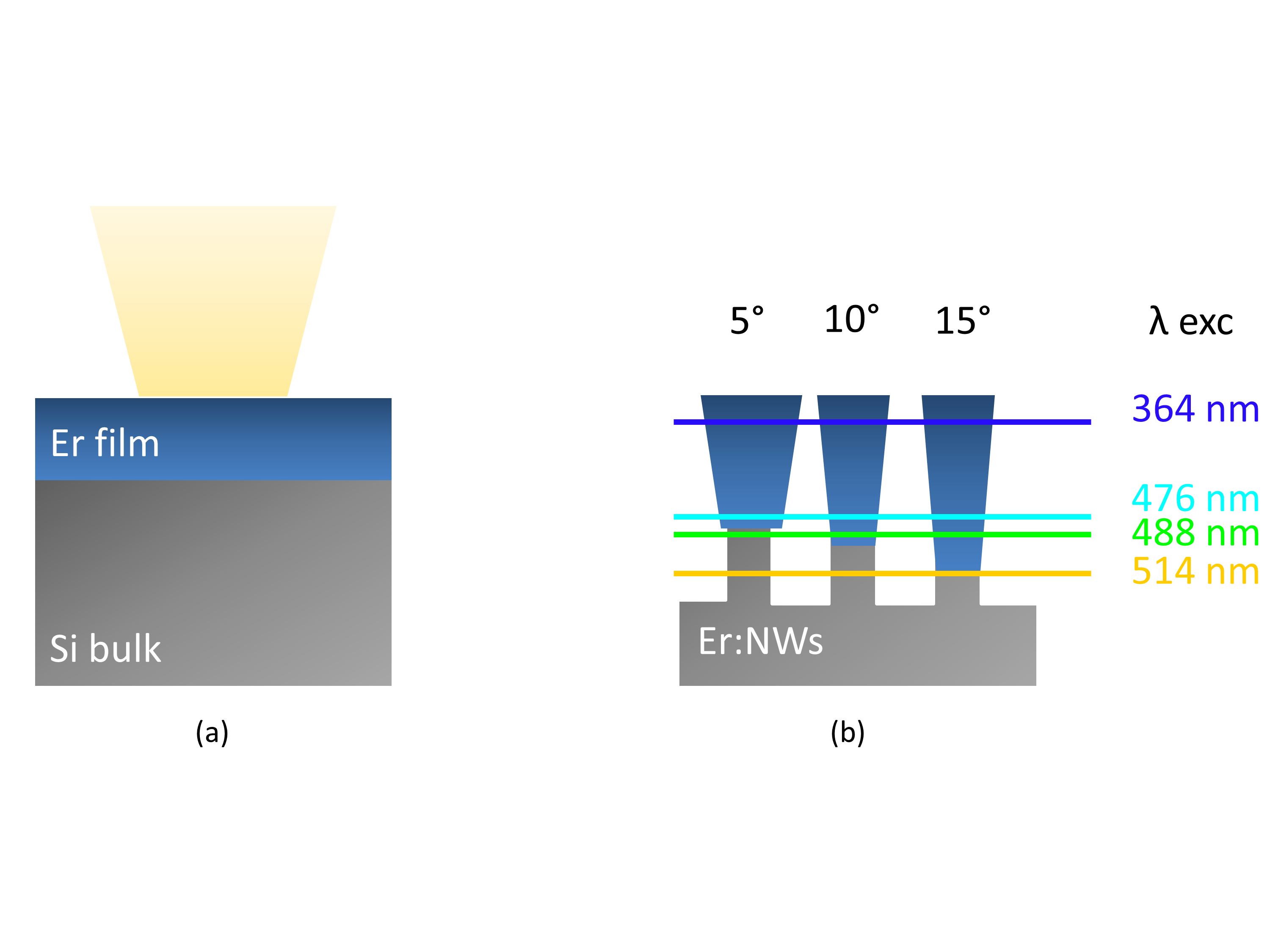


Figure S5: **Excitation scheme for the calculation of the excited Er fraction:** Schematic depicting the different Er distributions onto Si bulk and Si NWs. As described, all the available emitting centers are effectively illuminated at all the considered excitation wavelengths for the Er deposition onto Si bulk. Conversely, a different amount of Er is probed at the considered excitation onto Si NWs, as reported in table S2.

In order to estimate the portion of Er ions effectively probed at each excitation wavelength the light penetration depth δp (inelastic length) should be considered. This parameter accounts for the amount of Er emitters that effectively illuminated, defining the maximum length illuminated along the NWs profile (δ_p_) by considering that the light absorbed from the Si NWs slab cannot be scattered from the array nor absorbed by the Er centers. The table S2 summarized the penetration depths δ_p_ for each excitation wavelength (λ_exc_), obtained considering the absorption from about 40% of silicon as measured from the structural investigations. The different Er profiles as a function of deposition angles were also taken into account by considering that the total Er amount is the same for all the deposition angles. These different profiles as a function of the depth are confirmed by the filling factors increment for lower deposition angles and we estimated the maximum of Er profile (L_Er_) down to 1.5 µm, 1.25 µm and 1.10 µm at 5°, 10° and 5°, respectively. The effective Er fraction involved in photoluminescence was evaluated for each deposition by normalizing the light penetration depth for the decoration depths each excitation wavelength:

$$f_{Er}= \frac{L_{Er}}{\delta_{p}}$$

Indeed, the PL intensity ratio was scaled for the factor *f*_Er_ that accounts for the effectively probed Er concentration, as resumed in table S2.

We estimated a focal depth of about 1.4 µm at the center wavelength of 560 nm (length defining the portion from which the PL signal is optically collected). This focal depth value confirms that if excited, all the radiation can be collected through the objective to our detection system^7^.

|  |  | **Er decoration depth L_Er_**  **nm** | | |
| --- | --- | --- | --- | --- |
|  |  | **5°** | **10°** | **15°** |
|  |  | 1100 ± 123 | 1250 ± 141 | 1500 ± 178 |
| **Excitation**  **Wavelength λ_exc_** | **Light penetration**  **depth δ_p_** | **f_Er_** | | |
| **nm** | **nm** | **5°** | **10°** | **15°** |
| 364 | 27.5 ± 0.6 | 0.03 ± 0.01 | 0.02 ± 0.007 | 0.02 ± 0.007 |
| 476 | 1050 ± 21 | 0.95 ± 0.13 | 0.84 ± 0.12 | 0.70 ± 0.10 |
| 488 | 1190 ± 24 | 1.00 ± 0.14 | 0.95 ± 0.13 | 0.79 ± 0.11 |
| 514 | 1665 ± 33 | 1.00 ± 0.14 | 1.00 ± 0.14 | 1.00 ± 0.14 |

Table S2: **Optical parameters table.** Resuming table of the optical parameters used for the evaluation of the PL enhancement for each deposition angle: the maximum decoration depth (L_Er_) obtained from structural measurements, the fraction of Er emitting centers effectively illuminated onto Si NWs (*f*_Er_) and the light penetration depth calculated for each excitation wavelength considering the absorption from the Si NWs slab (composed of a 40 % of silicon).

According to the previous consideration, we estimated the enhancement factor for the 560 nm Er emission band per each titled deposition from the ratio of the Er integrated intensity peak onto Si NW versus the one on Si bulk scaled for the effective Er emitting centers, according to the equation (1) of the main text: $EF=\frac{I_{NWs}}{I_{film}} f_{Er\%}$.

The EF uncertainty was calculated by propagating the experimental errors from eq. 1 of the PL intensity ratio from Er:Y2O3/NWs versus Er:Y2O3 film on Si bulk (about 7% for each integrated peak), and the decoration length L_Er_ affected by EDX profilometry uncertainty (about 12% of the length). All the measured enhancement factors and their respective uncertainties are resumed in table S3.

| **EFs** | | |
| --- | --- | --- |
| **5°** | **10°** | **15°** |
|  |  |  |
| 45.7 ± 4.6 | 74.9 ± 7.5 | 125.8 ± 12.6 |
| 2.7 ± 0.3 | 6.9 ± 0.7 | 9.0 ± 0.9 |
| 1.4 ± 0.1 | 4.1 ± 0.7 | 6.6 ± 0.7 |
| 1.1 ± 0.1 | 2.3 ± 0.2 | 5.6 ± 0.6 |
|  |  |  |

Table S3: **Enhancement factor table.** Er luminescence enhancement factors measured for each deposition angle.

All the optical parameters, such as scattering and excitation cross sections, are driven by the lacunarity (length scale dependent refractive index fluctuation) ^8,9^. Hence, the EF values are reported in a semi-log scale in order to give an estimate of the scattering cross-section for each sample.

**References**

1. Fazio, B. *et al.* Strongly enhanced light trapping in a two-dimensional silicon nanowire random fractal array. *Light Sci. Appl.* **5**, (2016).

2. Irrera, A. *et al.* Quantum confinement and electroluminescence in ultrathin silicon nanowires fabricated by a maskless etching technique. *Nanotechnology* **23**, 075204 (2012).

3. RefractiveIndex.INFO - Refractive index database. Available at: https://refractiveindex.info/. (Accessed: 23rd June 2020)

4. Aspnes, D. E. & Studna, A. A. Dielectric functions and optical parameters of Si, Ge, GaP, GaAs, GaSb, InP, InAs, and InSb from 1.5 to 6.0 eV. *Phys. Rev. B* **27**, 985–1009 (1983).

5. Malitson, I. H. Interspecimen Comparison of the Refractive Index of Fused Silica. *J. Opt. Soc. Am.* **55**, 1205 (1965).

6. Nigara, Y. Measurement of the Optical Constants of Yttrium Oxide. *Jpn. J. Appl. Phys.* **7**, 404–408 (1968).

7. Novotny, L. & Hecht, B. *Principles of nano-optics*. *Principles of Nano-Optics* (Cambridge University Press, 2006). doi:10.1017/CBO9780511813535

8. Xu, M. & Alfano, R. R. Fractal mechanisms of light scattering in biological tissue and cells. *Opt. Lett.* **30**, 3051 (2005).

9. Tang, J. *et al.* Calculation extinction cross sections and molar attenuation coefficient of small gold nanoparticles and experimental observation of their UV–vis spectral properties. *Spectrochim. Acta - Part A Mol. Biomol. Spectrosc.* **191**, 513–520 (2018).
